# Immunomodulatory effects of cathelicidin in the gut–brain axis: A novel link between mucosal immunity and neuroinflammation

**DOI:** 10.1113/EP093221

**Published:** 2026-01-06

**Authors:** Mehrdad Nourizadeh, Amir Arsalan Ghahari, Ehsan Zandi, Seyedeh Zeynab Rasouli, Shaghayegh Davari, Mobina Hoseinzadeh, Mir Alireza Nourazar

**Affiliations:** ^1^ Neurosciences Research Center Tabriz University of Medical Sciences Tabriz Iran; ^2^ School of Medicine Shiraz University of Medical Sciences Shiraz Iran; ^3^ Department of Basic Sciences, Tabriz Medical Sciences Islamic Azad University Tabriz Iran

**Keywords:** cathelicidin, gut–brain axis, intestinal barrier, microbiota, mucosal immunity, neuroinflammation, short‐chain fatty acid

## Abstract

Cathelicidins are evolutionarily conserved host defence peptides known for their dual antimicrobial and immunomodulatory functions. Among them, LL‐37 in humans and CRAMP in rodents have emerged as crucial regulators of both mucosal immunity and CNS inflammation. This review explores the emerging evidence that positions cathelicidins as key modulators of the gut–brain axis, a bidirectional communication network increasingly implicated in neuroinflammatory and neurodegenerative disorders. Drawing on a diverse body of animal and human research, we examine the multifaceted roles of cathelicidin in maintaining intestinal barrier integrity, shaping microbiota composition and regulating innate immune signalling. Particular attention is paid to how gut‐derived metabolites, such as short‐chain fatty acids and vitamin D, influence cathelicidin expression, with downstream consequences for both gastrointestinal and neural health. In the CNS, cathelicidin exhibits context‐dependent effects, acting as a neuroprotective modulator when derived from neurons, but exacerbating glial‐mediated inflammation when sourced from peripheral immune cells. This functional dichotomy underscores the importance of cellular origin, concentration and microenvironmental cues. Furthermore, we delineate how cathelicidin facilitates crosstalk between peripheral and central compartments, serving as both a local effector and a systemic messenger. Collectively, these insights support a reconceptualization of cathelicidin not merely as a passive antimicrobial peptide, but as an active molecular bridge between mucosal immunity and neuroinflammation, with promising implications for diagnostics and therapeutics targeting dysfunction of the gut–brain axis.

## Abstract (highlights)

- **What is the topic of this review?**
  We describe cathelicidin (LL‐37/CRAMP) as an immunomodulator of the gut–brain axis, including its roles in mucosal immunity, microbiota composition, epithelial barrier integrity, innate receptor signalling and CNS neuroinflammation linking the gut and the brain.
- **What advances does it highlight?**
  We highlight context‐dependent, source‐specific actions (neuronal protection vs. glial‐mediated inflammation); regulation by vitamin D and short‐chain fatty acids; signalling via formyl peptide receptor 2 (FPR2) and toll‐like receptors (TLRs); bidirectional gut–brain communication; and translational prospects for biomarkers and therapeutic modulation.

## 1 INTRODUCTION

Long regarded as separate physiological domains, the immune system and the nervous system are now more widely acknowledged to be intricately linked (Yang et al., 2020). The gut–brain axis [GBA; a dynamic, two‐way network that connects the gastrointestinal (GI) tract and the CNS via immune, endocrine, metabolic and neural pathways] is at the intersection of this neuroimmune crosstalk. In this framework, mucosal immunity becomes a crucial regulator of neural function and homeostasis, rather than simply a barrier defence mechanism (Fang et al., 2022). A variety of illnesses, including multiple sclerosis (MS), neurodegeneration, psychiatric syndromes and inflammatory bowel disease (IBD), have been linked to a disturbance of this equilibrium (Dübüş et al., 2023).

The function of innate immune mediators in preserving gut and brain homeostasis is being highlighted by an increasing amount of research. Among these, host defence peptides known for their antimicrobial activity, known as cathelicidins, have recently attracted notice for their strong immunomodulatory functions (Yang et al., 2020). Neutrophils, epithelial cells and, more recently, neural cells, such as astrocytes and neurons, express the only known human cathelicidin, LL‐37, and its murine counterpart, CRAMP (cathelin‐related antimicrobial peptide). Cathelicidins affect many immune functions, such as cytokine production, chemotaxis, inflammasome activation and tissue repair, in addition to their capacity to lyse pathogens (Bogdanov et al., 2023). Curiously, cathelicidins have become important players at both ends of the GBA. LL‐37 improves mucosal integrity, changes the composition of the microbiota and controls Toll‐like receptor (TLR) signalling in the GI tract (Fang et al., 2022). Cathelicidins are also expressed in the CNS, where they can affect neuroinflammation, glial reactivity and blood–brain barrier (BBB) permeability (Yan et al., 2025). A complex but manageable signalling landscape is highlighted by the molecular cues that control this dual‐site activity, especially those that come from regulatory molecules, such as vitamin D, and microbial metabolites, such as short‐chain fatty acids (SCFAs) (Dezun et al., 2024).

With a focus on its developing role as a molecular bridge across the GBA, this review summarizes the state of our understanding regarding the immunomodulatory roles of cathelicidin in the gut and brain. We seek to understand how cathelicidin might unify various pathological processes and provide new targets for therapeutic intervention by combining mechanistic insights from human and animal studies.

## 2 CATHELICIDIN STRUCTURE, REGULATION AND TISSUE DISTRIBUTION

**FIGURE 1:**
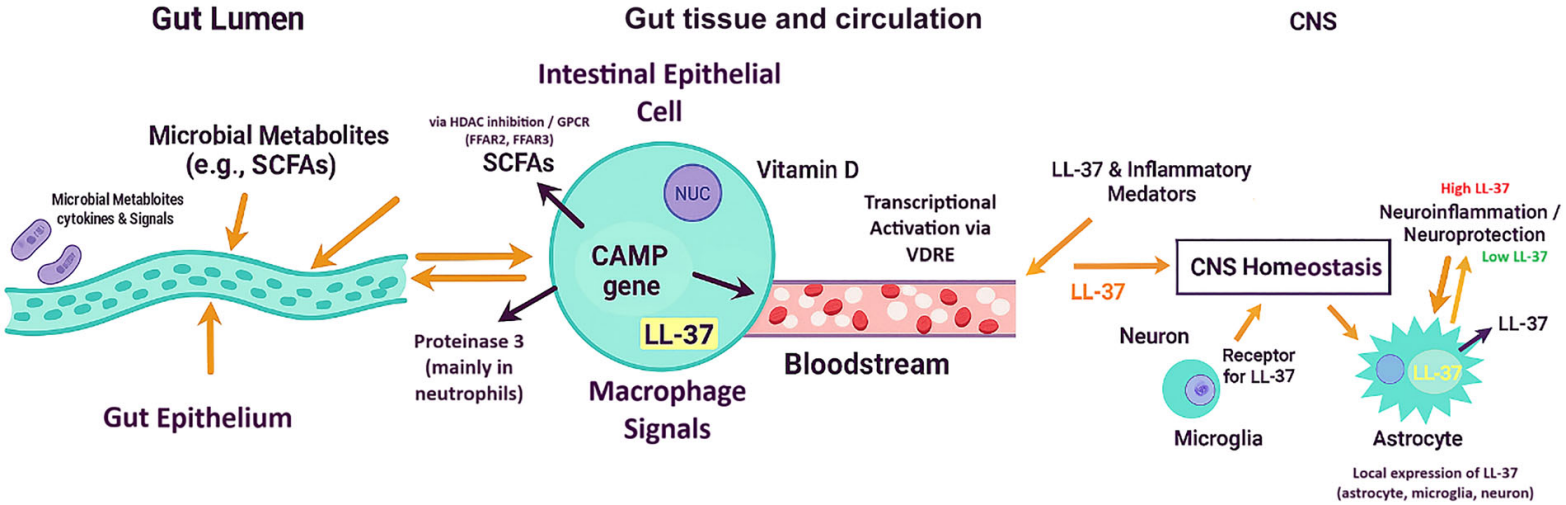
Schematic overview of cathelicidin‐mediated signalling along the gut–brain axis. Microbial metabolites in the gut lumen, particularly SCFAs, act on the gut epithelium and intestinal epithelial cells to induce *CAMP* gene transcription and LL‐37 production via HDAC and GPCR pathways, while vitamin D activates *CAMP* via VDRE in the same cells and in macrophages. LL‐37 released into the bloodstream integrates these gut‐derived signals and reaches the CNS, where neurons, microglia and astrocytes express receptors for LL‐37 and produce LL‐37 locally. Context‐dependent LL‐37 levels help to maintain CNS homeostasis at low concentrations but contribute to neuroinflammation and tissue injury when LL‐37 is excessively elevated. Abbreviations: *CAMP*, cathelicidin antimicrobial peptide gene (human); CNS, central nervous system; GPCR, G protein‐coupled receptor; HDAC, histone deacetylase; LL‐37, human cathelicidin antimicrobial peptide; SCFAs, short‐chain fatty acids; VDRE, vitamin D response element.

Cathelicidins are conserved host defence peptides found in birds, mammals and reptiles. In humans, the cathelicidin gene cathelicidin antimicrobial peptide (*CAMP*) encodes the inactive propeptide hCAP18, which is cleaved by serine proteases, such as proteinase 3, to generate the active peptide LL‐37; the murine orthologue *CRAMP* has a different sequence but similar immune functions (Tokajuk et al., 2022; Yang et al., 2020). LL‐37 is a 37‐amino‐acid peptide with two N‐terminal leucines that forms an amphipathic α‐helix, enabling membrane insertion, antimicrobial activity and immunomodulation through receptors and signalling pathways (Kulkarni et al., 2021). Cathelicidin expression is tightly regulated. Vitamin D activates *CAMP* via a vitamin D response element in macrophages and colonic epithelial cells, whereas microbial metabolites, SCFAs, such as butyrate, induce LL‐37 through histone deacetylase inhibition and G‐protein‐coupled receptors (GPCRs), including FFAR2 and FFAR3 (Aldekwer et al., 2022). Cathelicidin is produced by gut epithelial and immune cells; by keratinocytes and bronchial epithelial cells; and by neurons, astrocytes and microglia in the CNS; in a neuroinflammation model, Bhusal et al. (2022) showed that astrocyte‐derived CRAMP promotes glial activation (Bhusal, Nam, Seo, Rahman et al., 2022). Its actions are context dependent, with low levels supporting integrity and immune homeostasis, whereas high concentrations from infiltrating neutrophils intensify inflammation and tissue damage (Bogdanov et al., 2023). This regulated distribution supports cathelicidin as a signalling integrator along the GBA (Figure 1).

## 3 CATHELICIDIN IN MUCOSAL IMMUNITY: MECHANISMS IN THE GUT

Cathelicidin is a frontline immune effector in the gut that extends far beyond direct antimicrobial killing. LL‐37 in humans and its murine analogue, CRAMP, integrate microbial defence with barrier support and inflammatory control. They neutralize pathogens by binding to negatively charged bacterial membranes and disrupting their integrity, while modulating host signalling such that antimicrobial responses remain effective but not excessively damaging. Marin et al. (2017, 2019) showed that LL‐37 enhances intestinal epithelial resistance to *Salmonella enterica* by adjusting TLR4 signalling and interleukin‐1β (IL‐1β) production, thereby limiting NLRP3 inflammasome activation.

LL‐37 also strengthens the epithelial barrier by inducing mucins, such as MUC2 and MUC1, and by upregulating tight junction proteins, including occludin and ZO‐1, which reinforce paracellular sealing (Porter et al., 2021). These effects are crucial in infectious colitis, coeliac disease and IBD, where barrier breakdown amplifies inflammation; in a gluten‐induced enteropathy model, CRAMP deficiency increased permeability and exacerbated inflammation (Ren et al., 2021).

**FIGURE 2:**
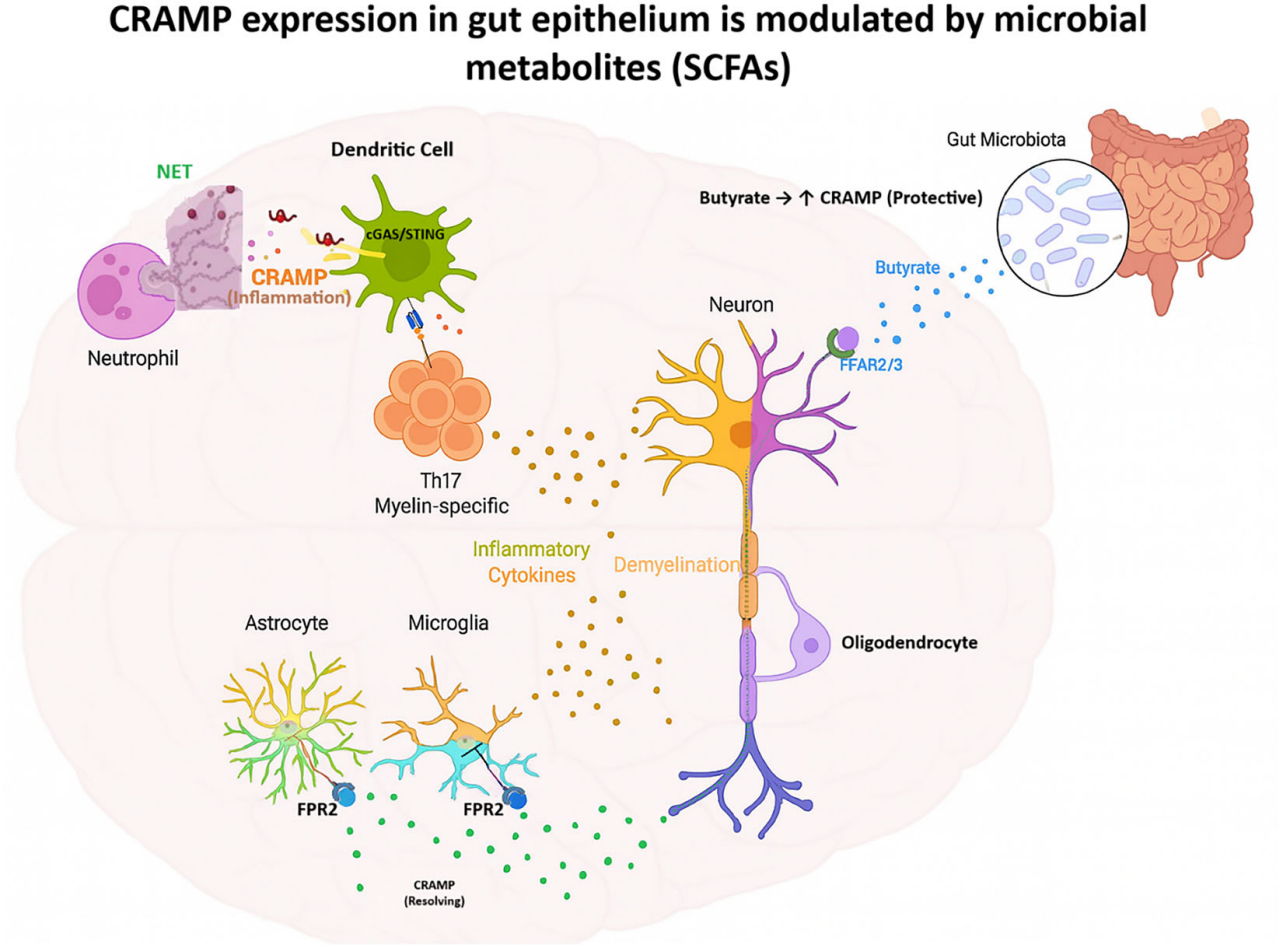
Schematic representation of the dual role of cathelicidin (LL‐37/CRAMP) in neuroinflammation and resolution within the gut–brain axis. In the intestinal compartment, microbial metabolites, such as butyrate, enhance CRAMP expression through FFAR2/3 signalling, contributing to protection of the epithelial barrier and immune homeostasis. Neutrophil‐derived CRAMP activates dendritic cells via the cGAS–STING pathway, promoting Th17 responses and secretion of inflammatory cytokines that drive demyelination and neuronal injury. Conversely, CRAMP binding to FPR2 on astrocytes and microglia supports anti‐inflammatory signalling and resolution of neuroinflammation. Together, these pathways highlight the bidirectional influence of microbial metabolism and cathelicidin activity in regulating gut–brain immune communication. Abbreviations: cGAS, cyclic GMP–AMP synthase; CRAMP, cathelin‐related antimicrobial peptide (murine cathelicidin); FFAR2/3, free fatty acid receptors 2/3; FPR2, formyl peptide receptor 2; LL‐37, human cathelicidin antimicrobial peptide; STING, stimulator of interferon genes; Th17, T helper 17

By regulating pattern recognition receptors (PRRs), cathelicidin can either dampen or amplify TLR pathways, suppressing TLR9‐driven inflammation in dendritic cells while enhancing TLR4 responses in epithelial cells (Kulkarni et al., 2021; Wu et al., 2020). It also shapes the microbiota by limiting pathobionts and supporting commensals (Fang et al., 2022). Through FPR2 and other GPCRs, LL‐37/CRAMP recruit neutrophils, monocytes and T cells, which can resolve infection or sustain chronic inflammation (Kulkarni et al., 2021; Popa et al., 2024). Studies in piglets and human epithelial cells show that cathelicidin analogues mitigate injury and tune TLR4–TLR9 balance (G. Kilari et al., 2025; Zhang et al., 2022). Collectively, these properties define cathelicidin as a context‐sensitive immune sentinel and an upstream regulator of gut–brain immune communication (Table 1; Figure 2).

**TABLE 1:** Select studies of cathelicidin in gut or CNS models

Reference	Model/system	Intervention	Main outcome	Mechanism/pathway
Marin et al. (2019)	T84 colonic epithelial cells	LL‐37 treatment versus knockdown + *Salmonella typhimurium*	LL‐37 restored ZO‐1 and reduced invasion; knockdown impaired TLR4/IL‐1β response	TLR4 modulation; cytokine regulation
G. Kilari et al. (2025)	T84 colonic epithelial cells	LL‐37 + lipopolysaccharide versus LL‐37 + CpG (TLR9)	LL‐37 enhanced TLR4/IL‐1β and IL‐8 (CXCL8); suppressed TLR9 response	Selective TLR4 ↑/TLR9 ↓; IL‐8 upregulation; immune fine‐tuning
Ren et al. (2021)	Gluten‐induced enteropathy (mouse)	*CRAMP* ^−/−^ versus CRAMP; microbiota profiling	CRAMP restored gut barrier; LasB protease degraded CRAMP	Barrier repair; CRAMP degradation by bacteria
Zhang et al. (2019)	Human and mouse gut	Functional role of LL‐37/CRAMP	Promoted mucosal integrity, epithelial renewal and MUC1/2 expression	MAPK pathway; mucin gene upregulation
Verma et al. (2024)	EAE model; neurons/glia	Neutrophil versus neuronal CRAMP; butyrate	Neutrophil‐CRAMP worsened EAE (Th17); neuronal CRAMP protective; butyrate increased neural CRAMP	FPR2 dual role; FFAR2/3–SCFA–CRAMP axis
Lee et al. (2015)	Human astrocytes and microglia	LL‐37 exposure	LL‐37 induced IL‐1β, IL‐6, IL‐8, CCL2 release	p38 MAPK and nuclear factor‐κB activation
Bhusal, Nam, Seo, Rahman et al. (2022)	EAE mouse; MS tissues	CRAMP injection versus knockdown	CRAMP elevated in astrocytes; injection worsened demyelination; knockdown reduced glial response	FPR2–STAT3 activation in microglia

Abbreviations: CNS, central nervous system; LL‐37, human cathelicidin antimicrobial peptide; CRAMP, cathelin‐related antimicrobial peptide (murine cathelicidin); LPS, lipopolysaccharide; CpG, cytosine–phosphate–guanine (TLR9 ligand); TLR, toll‐like receptor; ZO‐1, zonula occludens‐1; IL, interleukin; CXCL8, C‐X‐C motif chemokine ligand 8 (IL‐8); CCL2, C‐C motif chemokine ligand 2; MAPK, mitogen‐activated protein kinase; NF‐κB, nuclear factor kappa B; EAE, experimental autoimmune encephalomyelitis; MS, multiple sclerosis; Th17, T helper 17; FPR2, formyl peptide receptor 2; STAT3, signal transducer and activator of transcription 3; FFAR2/3, free fatty acid receptors 2/3; SCFA, short‐chain fatty acid; BBB, blood–brain barrier; CAMP, cathelicidin antimicrobial peptide gene (human); CNS, central nervous system; GPCR, G protein‐coupled receptor; HDAC, histone deacetylase; LL‐37, human cathelicidin antimicrobial peptide; SCFAs, short‐chain fatty acids; VDRE, vitamin D response element.

## 4 GUT‐DERIVED SIGNALS REGULATING Cathelicidin EXPRESSION

A complex web of systemic and local cues regulates the expression and activity of cathelicidin in the GI tract. Among these, inflammatory mediators, nutritional variables and microbial metabolites are important in determining the availability and functionality of cathelicidin. To comprehend fully how cathelicidin connects the gut environment to systemic and neuroimmune regulation, it is essential to comprehend these upstream signals (Fabisiak et al., 2024; Holani et al., 2020).

### 4.1 SCFAs and microbial metabolites

The gut microbiota ferments dietary fibre into SCFAs, notably butyrate, acetate and propionate. Butyrate strongly induces LL‐37 in neurons and colonic epithelial cells by inhibiting histone deacetylases (HDAC) and activating *CAMP* transcription (Bai et al., 2020; Liu et al., 2023). SCFAs also signal through FFAR2 and FFAR3 on epithelial and immune cells to modulate cathelicidin expression (Carbone et al., 2022). In mice, oral butyrate increases CRAMP in the gut and the CNS, reduces neuroinflammation, strengthens the BBB, and these benefits are blunted in CRAMP‐deficient animals (Elfadil et al., 2023; Saadh et al., 2025).

### 4.2 Vitamin D signalling

**FIGURE 3:**
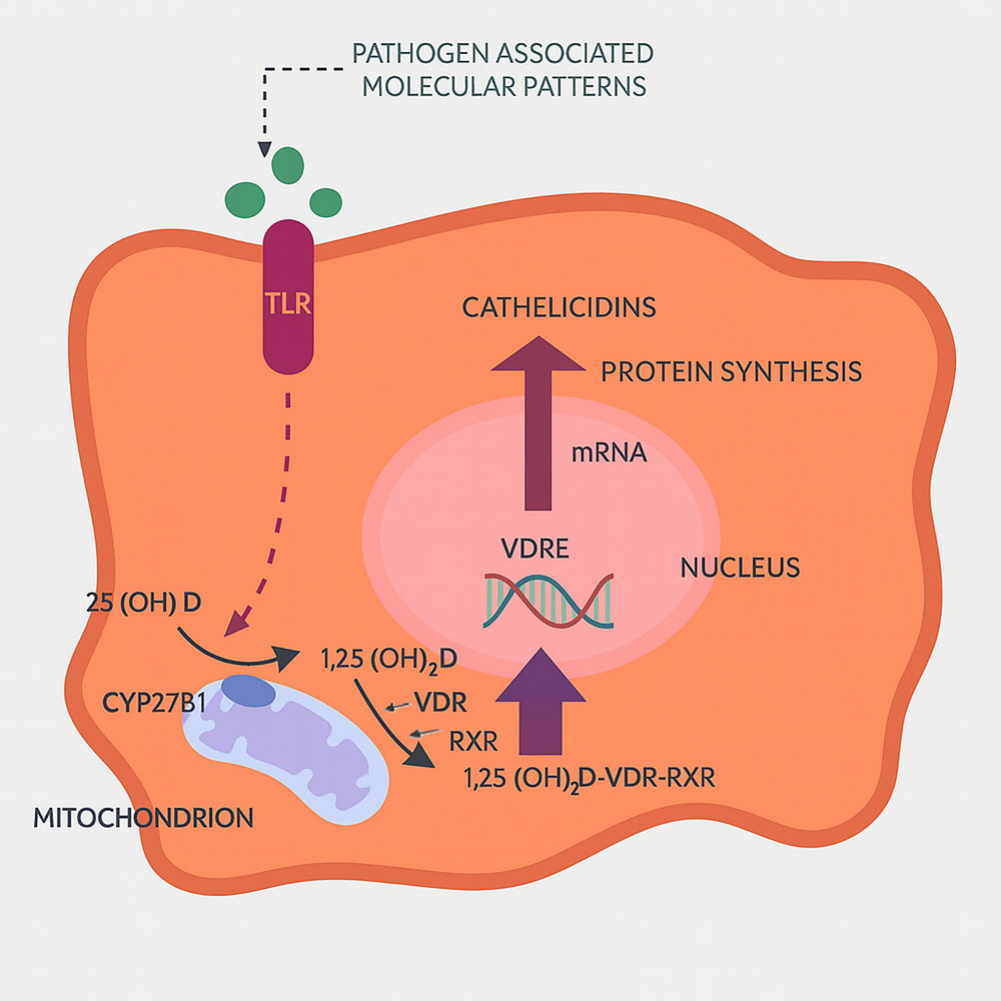
Vitamin D signalling pathway leading to cathelicidin synthesis. TLR activation by PAMPs induces CYP27B1 expression, converting 25(OH)D to 1,25(OH)_2_D, which forms a VDR‐RXR complex. This complex activates VDRE in the nucleus, promoting cathelicidin transcription and protein synthesis. Abbreviations: 1,25(OH)2D, 1,25‐dihydroxyvitamin D; 25(OH)D, 25‐hydroxyvitamin D; CYP27B1, cytochrome P450 family 27 subfamily B member 1; PAMPs, pathogen‐associated molecular patterns; RXR, retinoid X receptor; TLR, toll‐like receptor; VDR, vitamin D receptor; VDRE, vitamin D response element.

One well‐established endocrine pathway for regulating mucosal immunity is the vitamin D–cathelicidin axis. When vitamin D is converted to its hormone form (1,25‐dihydroxyvitamin D_3_), it attaches itself to the vitamin D receptor (VDR), which then uses vitamin D response elements (VDREs) to transactivate the *CAMP* gene directly (Chung et al., 2020). The intestinal epithelium, skin and immune cells are among the tissues that share this mechanism. Vitamin D supplementation improved epithelial barrier function and raised LL‐37 levels in colonic organoid models (Fabisiak et al., 2024). Furthermore, in patients with Crohn's disease and ulcerative colitis, clinical research has demonstrated associations between faecal LL‐37 concentrations, serum vitamin D levels and the severity of the disease. These results highlight how vitamin D can modulate gut‐derived cathelicidin in an immunonutritional manner (Chung et al., 2020; Figure 3).

### 4.3 Cytokines and PRRs

The production of LL‐37 can be either upregulated or repressed by inflammatory cytokines, such as IL‐1β, tumour necrosis factor‐α (TNF‐α) and interferon‐γ, depending on the cell type and stimulus. For example, IL‐1β increases nuclear‐κB and MAPK signalling to induce cathelicidin in intestinal epithelial cells (Jiang et al., 2020), whereas TNF‐α exhibits more variable effects depending on the presence of co‐stimulatory signals (Ribeiro et al., 2020). PRRs also play a part. TLR2 and TLR4 activation can work in concert with SCFAs or vitamin D to increase *CAMP* (human) or *Camp* (mouse) gene transcription, thereby elevating LL‐37/CRAMP peptide levels(A. Bleakley et al., 2021). Notably, LL‐37 itself regulates PRR activity, creating a feedback loop, in which the expression of its own immune effectors is influenced by microbial detection (A. Bleakley et al., 2021; Jiang et al., 2020).

### 4.4 Dietary and hormonal influences

It has been reported that certain dietary components, including omega‐3 fatty acids, curcumin and polyphenols, indirectly alter LL‐37 expression through anti‐inflammatory and antioxidant pathways. Cathelicidin transcription can also be influenced by glucocorticoids and sex hormones, such as oestrogen, although the exact mechanisms in gut tissues are less clear (Yang et al., 2020).

In conclusion, the production of cathelicidin in the gut is dynamic and responsive to microbial, nutritional and environmental stimuli. Particularly noteworthy as central regulators are SCFAs and vitamin D, which can connect dietary status and microbiota composition to mucosal immunity (Bleakley et al., 2021). The integration of gut environmental cues with systemic and CNS immune responses, the cornerstone of gut–brain communication, is facilitated by these upstream modulators (A. Aidoukovitch et al., 2024).

## 5 CATHELICIDIN EXPRESSION AND FUNCTION IN THE CENTRAL NERVOUS SYSTEM

Despite being thought of as a peripheral host defence peptide, cathelicidin has been found to have a complex, context‐dependent impact on neuroimmune processes in the CNS (Bhusal, Nam, Seo, Rahman et al., 2022). Cathelicidin is a crucial, albeit underappreciated, participant in CNS immune regulation owing to its expression in neural tissues, such as neurons, astrocytes and microglia (Bhusal, Nam, Seo, Lee et al., 2022), and its capacity to regulate both protective and pathological neuroinflammation (Amoriello & Ballerini, 2025).

### 5.1 Sources and regulation in the CNS

Cathelicidin is expressed in the CNS in both physiological and pathological conditions (Spurgat & Tang, 2022). In rodents and humans, neurons can produce CRAMP or LL‐37 in response to stress, infection or inflammation (Bhusal, Nam, Seo, Rahman et al., 2022). Pro‐inflammatory signals, such as lipopolysaccharide, IL‐1β and microbial metabolites, upregulate cathelicidin in astrocytes and microglia (Xingi et al., 2023). Bhusal et al. (2022) showed that inflammatory cues markedly increase astrocytic CRAMP, which enhances glial activation and neurotoxicity in vitro and in vivo (Bhusal, Nam, Seo, Rahman et al., 2022). Similar to peripheral tissues, vitamin D and microbial SCFAs regulate LL‐37 in neural cells. Butyrate has been reported to reduce microglial activation and elevate CRAMP expression in hippocampal neurons, suggesting that microbial metabolites can modulate CNS immunity through host defence peptide regulation (Triviño & Von Bernhardi, 2021).

### 5.2 Functional duality: Neuroprotection versus neuroinflammation

The effects of cathelicidin in the CNS depend strongly on its cellular source and concentration (Verma et al. 2024). Neuron‐derived cathelicidin appears neuroprotective, because low CRAMP levels enhance synaptic plasticity, support neuronal survival and limit oxidative stress (Bhusal, Nam, Seo, Rahman et al., 2022). In contrast, high cathelicidin concentrations from reactive glia or infiltrating neutrophils can disrupt the BBB, drive neuroinflammation and promote neuronal injury (Bhusal, Nam, Seo, Rahman et al., 2022). This duality is likely to reflect receptor‐specific signalling. LL‐37 activates receptors such as FPR2, P2X7 and TLR9, whose expression patterns differ among CNS cell types (Dabravolski et al., 2024). In astrocytes, LL‐37–FPR2 signalling triggers STAT3 and ERK1/2 pathways, leading to GFAP upregulation and the release of pro‐inflammatory cytokines, including IL‐6 and TNF‐α (Bhusal, Nam, Seo, Rahman et al., 2022). In neurons, however, FPR2 activation might initiate survival and anti‐apoptotic pathways.

### 5.3 Impact on the BBB and immune cell recruitment

Cathelicidin also modulates BBB integrity (Bhusal, Nam, Seo, Rahman et al., 2022). In physiological conditions, LL‐37 supports tight junctions and endothelial homeostasis (Verma et al., 2024). In inflammation, however, it upregulates ICAM‐1 and VCAM‐1, increases endothelial permeability and facilitates leucocyte transmigration, with higher LL‐37 being correlated with neutrophil influx and CNS damage in bacterial meningitis models (Bhusal, Nam, Seo, Rahman et al., 2022; Dabravolski et al., 2024). Through GPCR signalling, LL‐37 attracts neutrophils and monocytes, which are beneficial during infection but can exacerbate autoimmune or sterile CNS inflammation, such as MS or Alzheimer's disease (Verma et al., 2024).

## 6 GUT‐TO‐BRAIN COMMUNICATION: PERIPHERAL CATHELICIDIN SIGNALLING INTO THE CNS

Peripheral immune signals that originate in the GI tract can impact CNS function through the GBA, a highly integrated communication system (Alzubide & Alhalafi, 2024). Cathelicidin is one of these immune messengers, which has shown promise as a molecular bridge that converts signals from mucosal surfaces into either neuroprotective or neuroinflammatory reactions in the brain (Bhusal, Nam, Seo, Rahman et al., 2022). The mechanisms through which gut‐derived cathelicidin affects the CNS remotely are examined in this section.

### 6.1 Systemic circulation and immune crosstalk

When microbial dysbiosis, infection or epithelial stress occurs, gut‐derived LL‐37 can enter the circulation (Kilari et al., 2025). In blood, it acts on immune tissues, endothelial cells and monocytes to promote migration, dendritic cell maturation and the release of IL‐6, IL‐1β and TNF‐α, which can modulate CNS inflammation (Bogdanov et al., 2023). Plasma LL‐37 is increased in gut‐leakage states, such as colitis and gluten sensitivity (Fang et al., 2022). Through cytokine cascades and endothelial activation, elevated LL‐37 can alter BBB permeability or act directly on CNS‐resident cells, in part by regulating ICAM‐1 and claudin‐5 expression in BBB endothelial cells (Shih et al., 2023).

### 6.2 Vagal and neuroimmune reflex pathways

Gut‐derived immune signals can also be transmitted to the brain through neural circuits, especially the vagus nerve, in addition to humoral transmission. Despite not being a neurotransmitter, LL‐37 can affect vagal afferents indirectly, by encouraging the release of neuroactive cytokines and by interacting with enteroendocrine cells that release neuromodulatory peptides such, as glucagon‐like peptide‐1 and cholecystokinin (Ramadan et al., 2025). According to recent research, LL‐37 might also modify afferent vagal tone by changing the composition of the gut microbiota and microbial metabolites, especially SCFAs, which affect vagal activation thresholds. Even in the absence of direct transport across the BBB, cathelicidin can participate in gut‐to‐brain signalling through this indirect mechanism (Jameson et al., 2025).

### 6.3 Microbiota‐mediated induction of CNS cathelicidin

The control of cathelicidin expression in distant tissues by the microbiota represents another key gut–brain communication route. Microbial SCFAs, particularly butyrate, can induce CRAMP expression in neurons and glia (Aggarwal et al., 2023), providing a mechanism through which changes in gut microbial composition propagate to CNS cathelicidin levels and neuroimmune tone. In murine neuroinflammation models, dietary butyrate or vitamin D increases brain CRAMP and dampens glial activation, consistent with a relay from microbiota to gut epithelium to brain (Bhusal, Nam, Seo, Rahman et al., 2022).

Overall, peripheral cathelicidin functions as a conduit through which mucosal immune signals influence the CNS. The gut emerges as a distant yet powerful regulator of brain health, with gut‐derived LL‐37 shaping the neuroinflammatory milieu via direct transport, modulation of barrier properties and engagement of neuroimmune signalling circuits (Postolache et al., 2020) (Table 2).

**TABLE 2:** Multilevel pathways and regulatory mechanisms of cathelicidin (LL‐37/CRAMP) in gut‐to‐brain communication.

Source/trigger	Target tissue or cell Type	Effect on LL‐37/CRAMP	Mechanism or pathway	Functional outcome in CNS	Key references
**Short‐chain fatty acids (butyrate, acetate)**	Colonic epithelial cells, hippocampal neurons	↑ *CAMP/Camp* transcription (↑ LL‐37/CRAMP)	HDAC inhibition; FFAR2/3 activation	Enhanced gut barrier; reduced neuroinflammation via CNS CRAMP expression	Aggarwal et al. (2023); Bhusal, Nam, Seo, Rahman et al. (2022); Siednamohammeddeen et al. (2022)
**Vitamin D (1,25‐dihydroxy D_3_)**	Intestinal epithelium, immune cells, CNS	↑ LL‐37 via VDR binding	*CAMP* gene activation via VDRE	Anti‐inflammatory effects; reduced glial activation	Bhusal, Nam, Seo, Rahman et al. (2022); Siednamohammeddeen et al. (2022)
**Cytokines (IL‐1β, TNF‐α)**	IECs, microglia, astrocytes	↑ or ↓ LL‐37 (context dependent)	Nuclear factor‐κB, MAPK pathway modulation	Bidirectional neuroimmune modulation	A. Bhusal, Y. Nam, D. Seo, M. H. Rahman, et al. (2022); Bogdanov et al. (2023); Kilari et al. (2025)
**TLRs (TLR2/4/9)**	IECs, glia	Synergistic with SCFAs/vitamin D	PRR activation + LL‐37 feedback loop	Enhanced pathogen defence, controlled neuroinflammation	Jameson et al. (2025); Ramadan et al. (2025)
**Circulating LL‐37**	Blood–brain barrier endothelial cells	↑ ICAM‐1, claudin‐5; permeability change	Direct endothelial interaction + cytokine priming	Immune cell infiltration into CNS; blood–brain barrier modulation	Ho et al. (2020b); Shih et al. (2023)
**Monocyte/dendritic cell activation**	Peripheral immune cells → CNS	Indirect ↑ CNS cytokine levels	LL‐37‐induced maturation and migration	Promotes or dampens CNS inflammation	Bogdanov et al. (2023); Fang et al. (2022); Kilari et al. (2025)
**Enteroendocrine signalling/vagal reflex**	Glucagon‐like peptide‐1/cholecystokinin cells → vagus nerve → brainstem	Indirect vagal tone modulation	SCFA–LL‐37–cytokine–vagus axis	Adjusted HPA tone; neuroimmune tuning	Jameson et al. (2025); Ramadan et al. (2025)
**Neuronal versus glial cathelicidin**	CNS neurons versus glia	Neuronal CRAMP: protective; glial CRAMP: inflammatory	Receptor‐specific: FPR2, STAT3, ERK1/2	Synaptic plasticity versus astrogliosis	A. Bhusal, Y. Nam, D. Seo, M. H. Rahman et al. (2022); Aggarwal et al. (2023)
**Microbiota via LL‐37 modulation**	Gut microbes → short‐chain fatty acids → CNS	Indirect ↑ CRAMP in CNS	Microbial metabolite‐driven upregulation	Microbiota‐programmed neuroimmune suppression	Bhusal, Nam, Seo, Rahman et al. (2022); Siednamohammeddeen et al. (2022)

Abbreviations: SCFAs, short‐chain fatty acids; LL‐37, human cathelicidin antimicrobial peptide; CRAMP, cathelin‐related antimicrobial peptide (murine cathelicidin); HDAC, histone deacetylase; FFAR2/3, free fatty acid receptors 2/3; VDR, vitamin D receptor; VDRE, vitamin D response element; IL‐1β, interleukin‐1 beta; TNF‐α, tumor necrosis factor alpha; NF‐κB, nuclear factor kappa B; MAPK, mitogen‐activated protein kinase; TLRs, toll‐like receptors; PRR, pattern recognition receptor; BBB, blood–brain barrier; ICAM‐1, intercellular adhesion molecule 1; CNS, central nervous system; GLP‐1, glucagon‐like peptide‐1; CCK, cholecystokinin; HPA, hypothalamic–pituitary–adrenal; FPR2, formyl peptide receptor 2; STAT3, signal transducer and activator of transcription 3; ERK1/2, extracellular signal‐regulated kinase 1/2; IECs, intestinal epithelial cells.

## 7 BRAIN‐TO‐GUT FEEDBACK: CENTRAL NERVOUS SYSTEM‐DERIVED CATHELICIDIN AND PERIPHERAL IMMUNITY

Although gut‐derived signals that affect the brain have been emphasized most strongly, the reverse direction of the GBA is increasingly recognized as an important feedback pathway. During neuroinflammatory states, LL‐37/CRAMP produced by neurons and glial cells can appear in blood and CSF and is associated with enhanced leucocyte trafficking and systemic cytokine responses, indicating that CNS‐derived cathelicidin can modulate peripheral immunity (Chung et al., 2020; Masanetz et al., 2022). Neuroinflammation also drives sympathetic outflow and activation of the hypothalamic–pituitary–adrenal axis, which, in turn, shapes intestinal immune tone and barrier function (Masanetz et al., 2022). Experimental data suggest that cathelicidin might influence autonomic circuits directly by modulating the excitability of sympathetic neurons and indirectly by affecting enteric glial signalling, epithelial secretion, motility and tight junction organization, with higher brain CRAMP levels being correlated with immune cell infiltration and altered junctional architecture in colonic tissue (Kilari et al., 2025).

These systemic and neural outputs converge on the intestinal environment, where LL‐37 can modify microbial communities and contribute to dysbiosis, reduced bacterial diversity and expansion of pathobionts in susceptible settings (Fang et al., 2022; Kilari et al., 2025). A plausible feedback loop therefore emerges: dysbiosis and mucosal inflammation amplify CNS activation, which increases CNS‐derived cathelicidin; this, in turn, further stimulates peripheral immune responses and reshapes the gut milieu (Fang et al., 2022). In this framework, CNS‐derived LL‐37 is not confined to local neural effects but functions as both an effector and a mediator in descending brain‐to‐gut communication, helping to sustain bidirectional immune dialogue along the GBA (Chung et al., 2020; Masanetz et al., 2022).

## 8 EXPERIMENTAL MODELS, TOOLS AND TRANSLATIONAL EVIDENCE

Cathelicidin function along the GBA has been explored using genetic, pharmacological and biomarker‐based approaches. In mice, *Camp* knockout (*Camp* ^−/−^) and CRAMP overexpression or supplementation establish causal links with microbiota composition, epithelial permeability, dextran sodium sulphate colitis severity and neuroinflammatory phenotypes, such as experimental autoimmune encephalomyelitis and lipopolysaccharide‐induced glial activation (Holani et al., 2020; Wu et al., 2020; Zhai et al., 2023). LL‐37/CRAMP expression is typically quantified by qPCR for *CAMP/Camp* transcripts and by Western blotting, immunohistochemistry or ELISA to assess peptide levels in the gut, brain, serum and CSF, often together with multiplex cytokine profiling (Bhusal, Nam, Seo, Lee et al., 2022; Aldekwer et al., 2022). in vitro, intestinal epithelial, astrocyte and microglial cultures permit mechanistic studies on barrier integrity, FPR2‐dependent signalling and *CAMP* promoter regulation by SCFAs, vitamin D and TLR ligands, with synthetic LL‐37 analogues, such as cathelicidin‐WA, used as pharmacological probes (Aldekwer et al., 2022; Amagai et al., 2023). Translational work measures LL‐37 in serum, faecal and CSF samples from patients with IBD, MS, Alzheimer's disease and infectious conditions, increasingly using mass spectrometry, and evaluates LL‐37 mRNA in peripheral blood mononuclear cells as a marker of systemic immune activation (Majewski, Kozłowska et al., 2018; Zhang et al., 2023). Together, these tools link cathelicidin biology in the gut and brain to clinically measurable biomarker signatures along the GBA.

Across these models, convergent findings support cathelicidin as a key mediator of gut–brain neuroimmune crosstalk. *CAMP* ^−/−^ mice develop more severe colitis with barrier failure, leucocyte influx and exaggerated cytokine production, whereas exogenous or overexpressed CRAMP partly restores epithelial integrity and attenuates inflammation (Cavalcante et al., 2020; Cobo et al., 2017). In neuroinflammatory models, altered glial CRAMP or LL‐37 is associated with BBB disruption and glial activation, with astrocyte‐derived CRAMP aggravating disease via FPR2–ERK1/2 signalling (Bhusal, Nam, Seo, Rahman et al., 2022). Microbiota‐focused studies show that butyrate and other SCFAs increase CRAMP in the gut and the brain, improve anxiety‐ and memory‐related behaviours and reduce pro‐inflammatory mediators; germ‐free or antibiotic‐treated animals exhibit reduced cathelicidin expression and heightened neuroinflammation, which can be reversed by butyrate or microbial reconstitution (Firoozi et al., 2025; Liu et al., 2021).

Human data, although still limited, point in a similar direction. In IBD, serum and faecal LL‐37 concentrations rise during active disease and are correlated with endoscopic and clinical severity (Firoozi et al., 2025). LL‐37 is detectable in CSF from patients with Alzheimer's disease and MS, where it is associated with neuroinflammatory markers and cognitive decline (Liu et al., 2021). Pilot interventions that increase LL‐37, such as vitamin D supplementation, suggest modest reductions in systemic inflammation and possible mood or cognitive benefits in chronic inflammatory states (Elfadil et al., 2023). Translation to clinical practice is complicated by species differences between LL‐37 and CRAMP, heterogeneous dosing strategies and incomplete understanding of receptor specificity, underscoring the need for controlled trials that combine vitamin D, SCFA‐based approaches or LL‐37 analogues with longitudinal multi‐omics, neuroimaging and computational modelling (Elfadil et al., 2023; Firoozi et al., 2025; Liu et al., 2021).

## 9 DISCUSSION

Cathelicidin has recently emerged as a pivotal molecular bridge between mucosal and neural immunity along the GBA. Integrating molecular, cellular, animal and translational data, current evidence supports the view that LL‐37/CRAMP functions as a bidirectional immune mediator that can synchronize inflammatory signalling between the GI tract and the CNS (Bhusal, Nam, Seo, Lee et al., 2022). Cathelicidin is now recognized as a dynamic modulator of neuroimmune crosstalk, with protective or pathological effects that depend on the tissue context, receptor engagement and peptide concentration.

Functionally, cathelicidin shapes mucosal defence by tuning TLR signalling, preserving epithelial barrier integrity and limiting pathogen translocation, while also influencing microglial and astrocytic responses in the CNS. This context‐dependent duality allows LL‐37 either to aggravate inflammation and BBB disruption or to support neuronal survival and tissue repair. Environmental and microbial cues further refine this balance. SCFAs and vitamin D regulate cathelicidin expression in both intestinal and neural compartments, creating a biochemical link between diet, microbiota composition and neural outcomes (Bhusal, Nam, Seo, Rahman et al., 2022; Da Silva & Machado, 2017; Marin et al., 2019). These properties highlight the therapeutic appeal of targeting cathelicidin pathways in disorders that combine mucosal and neuroinflammatory dysfunction, including MS, Alzheimer's disease and IBD (Ho et al., 2020a; Kilari et al., 2025).

Key challenges remain. The threshold at which cathelicidin shifts from neuroprotective to neurotoxic activity is still undefined and is likely to shaped by glial priming, BBB status and systemic inflammation (Postolache et al., 2020). Human data are largely correlative, with additional heterogeneity from *CAMP* expression and vitamin D responsiveness (Aldekwer et al., 2022; Majewski, Agier et al., 2018; Wei et al., 2019). Receptor specificity is also unresolved, because LL‐37 signals through FPR2, P2X7 and TLR9 on diverse immune and neural cells. Moreover, structural and functional differences between murine CRAMP and human LL‐37 limit direct translation (Chen et al., 2018; Kilari et al., 2025). Future humanized and organ‐on‐chip models, coupled with longitudinal multi‐omics and neuroimaging, will be essential to define causal relationships within the gut–brain–cathelicidin axis and to guide safe, targeted interventions (Coorens et al., 2017; Postolache et al., 2020).

## 10 CLINICAL IMPLICATIONS

Recognition of cathelicidin as a molecular mediator connecting mucosal and neural immunity has important clinical implications. Circulating and tissue levels of LL‐37 provide accessible indicators of mucosal and neuroimmune activation. Elevated faecal LL‐37 is associated with mucosal ulceration, active disease flare and poorer therapeutic response in IBD, whereas CSF LL‐37 is correlated with neuroinflammatory load and cognitive decline in MS and Alzheimer's disease (Chen et al., 2018; Sun et al., 2016). Incorporating LL‐37 into multi‐analyte diagnostic panels might therefore improve monitoring of disorders along the GBA and offer a dynamic readout of responses to metabolic, microbial or pharmacological interventions (Burkes et al., 2019).

Therapeutically, moderate enhancement of cathelicidin activity through nutritional, microbial or pharmacological strategies can help to preserve epithelial integrity, attenuate cytokine‐driven inflammation and modulate glial responses in experimental models, with early clinical data suggesting symptomatic benefits in some chronic inflammatory conditions (Aidoukovitch et al., 2024; Aldekwer et al., 2022; Wei et al., 2019). Synthetic cathelicidin analogues, such as cathelicidin‐WA, which show increased stability and reduced cytotoxicity compared with native LL‐37, further support the feasibility of targeting this pathway as part of precision‐oriented approaches to GI and neuroimmune diseases (Burkes et al., 2019).

At the same time, LL‐37 represents a double‐edged mediator. Excessive or prolonged expression might intensify inflammation and glial activation in the CNS, raising the risk of tissue injury (Chen et al., 2018; Sun et al., 2016). Engagement of multiple receptors, including FPR2, P2X7 and TLR9, complicates receptor‐specific targeting and dosing (Bogdanov et al., 2023; Chen et al., 2018). Future investigations should account for inter‐individual variation in *CAMP* gene expression and responsiveness, refine the timing and route of therapeutic modulation and develop targeted delivery systems that limit off‐target and systemic toxicity (Wei et al., 2019). Well‐designed longitudinal human studies that integrate LL‐37 quantification with neuroimaging, immunological profiling and clinical outcomes will be crucial to define safe therapeutic windows and to determine whether cathelicidin‐cantered interventions can durably modify the course of GBA disorders (Burkes et al., 2019; Postolache et al., 2020).

## 11 CONCLUSION

Cathelicidin has emerged as a key molecular mediator within gut–brain communication. In humans, LL‐37 and its rodent counterpart, CRAMP, operate at the interface of mucosal immunity, microbiota regulation and neuroinflammation, integrating environmental, microbial and nutritional cues into coordinated immune responses. By influencing epithelial barrier integrity, microbial composition and both systemic and local immune signalling, cathelicidin contributes to both neuroprotective and neurotoxic processes in the CNS through effects on glial activation, BBB function and cytokine networks.

The evidence synthesized in this review supports a bidirectional feedback loop in which CNS‐derived cathelicidin shapes intestinal homeostasis and peripheral immunity, whereas gut‐derived cathelicidin modulates neuroimmune status. This recursive circuitry links cathelicidin to the pathophysiology of multiple disorders, including IBD, neurodegenerative diseases and microbiota‐related behavioural conditions. At the same time, the dual nature of the actions of cathelicidin, the limited availability of selective pharmacological tools and the scarcity of longitudinal human data underscore the need for further research.

Future work should prioritize mechanistic clarity in human‐relevant systems, including humanized models and organ‐on‐chip platforms, and should be complemented by carefully designed clinical studies that integrate cathelicidin measurements with imaging, immunological and behavioural outcomes. Overall, cathelicidin is not merely a peripheral antimicrobial peptide but a complex immunomodulator with far‐reaching implications for gut–brain communication and neuroimmune health. Elucidating and safely harnessing this pathway might open new therapeutic avenues at the intersection of immunology, neuroscience and gastroenterology.

## AUTHOR CONTRIBUTIONS

Mehrdad Nourizadeh, Amir Arsalan Ghahari and Mir Alireza Nourazar: Conceptualization, Writing—original draft, Writing—review & editing, Supervision. Ehsan Zandi, Seyedeh Zeynab Rasouli, Shaghayegh Davari, Mobina Hoseinzadeh: Conceptualization, Writing—original draft, Writing—review & editing. All authors approved the final version of the manuscript and agree to be accountable for all aspects of the work in ensuring that questions related to the accuracy or integrity of any part of the work are appropriately investigated and resolved. All persons designated as authors qualify for authorship, and all those who qualify for authorship are listed.

## CONFLICT OF INTEREST

None declared.

## FUNDING INFORMATION

None.

## Data Availability

Data sharing is not applicable to this article because no datasets were generated or analysed during the present study.
